# Personal, Political, Pedagogic: Challenging the Binary Bind in Archaeological Teaching, Learning and Fieldwork

**DOI:** 10.1007/s10816-016-9292-0

**Published:** 2016-08-24

**Authors:** Hannah Cobb, Karina Croucher

**Affiliations:** 1 Archaeology, University of Manchester, Manchester, M13 9PL UK; 2 Archaeological Sciences, University of Bradford, Bradford, BD7 1DP UK

**Keywords:** Assemblage theory, Pedagogy, Teaching and learning, Fieldwork, Diversity, Technology

## Abstract

In this paper, we consider how we can undercut the various binaries of gender and sexuality in archaeological practice and particularly in our teaching. We argue that taking an assemblage theory approach enables us to look at the multiplicity of identities of those practicing archaeology as different and intersecting assemblages that bring one another into being through their connections at different scales. In particular, we examine how this approach can be applied to archaeological pedagogy and how this in turn enables us to move away from modern binary distinctions about sex and gender identities from the ‘bottom up’, fostering an approach in our students that will then go on to be developed in professional practice.

## Introduction

It is a feminist mantra that the personal is political and that the political is personal. In academia, this is well recognised and the feminist critique centres around inequalities in research cultures, from the impact of parental leave to the glass ceilings in pay and promotions. This critique is often about the upper echelons of academia or at the very least focused on those with a doctorate and beyond. In contrast, we want to take a different approach in this paper and think about the binaries that bind our academic practice in other ways, in the less considered realm of pedagogy. We will demonstrate that if we want to strive for the culture change that will impact those in early or established academic careers, or indeed those beyond academia, in the broader archaeological profession, then we need to think about the students and about the cultures of teaching and learning which currently perpetuate the very binaries we hope to escape. To do this, we turn first to explore the available global demographics of professional archaeology, before exploring how these connect to diversity and pedagogy in the discipline.

## Archaeological Demographics

We are fortunate that the employment profile of archaeology is starting to be regularly examined. Consequently, we are able to draw on several key studies to examine the demographics of the discipline: the *Discovering the Archaeologists of Europe*: *Profiling the Profession* for the UK; the *Australian Archaeology in Profile* study; and the *American Archaeologists: A Profile* and the *Society for American Archaeology 2005 Salary Survey*. Whilst these focus on the Western world, combining their results here is the closest we have to a global picture of the profession.

Turning first to the data for Europe, the *Discovering the Archaeologists of Europe* labour market profiling exercise has been undertaken twice, in 2006–2008 and 2012–2014 (Aitchison 2009; Aitchison *et al.* 2014), whilst in the UK, this has been undertaken every 5 years since 1997–1998 in the form of the *Profiling the Profession* studies (Aitchison 1999; Aitchison and Edwards 2003, 2008; Aitchison and Rocks-Macqueen 2013), commissioned by the Chartered Institute for Archaeologists. These studies are taken, like a census, on a particular date and are completed by employers. They document all archaeologists working in the profession, from those in the commercial sector, to local and national government archaeologists, to academics and those in other organisations that do not fit neatly into the above categories (*e.g.* voluntary organisations, pressure groups and the media). In turn, this provides a very strong understanding of the gendered profile of the profession.

The most recent *Profiling the Profession* study (Aitchison and Rocks-Macqueen 2013), undertaken in 2012, presents statistics on gender that are, to some extent, very positive. There are more women than men in the profession under the age of 40, indicating that the gender gap is narrowing in UK archaeology. However, if we look beyond this, at other elements of the *Profiling the Profession* data on diversity, the picture is not so positive. Crucially, when age and gender are correlated, a more troubling pattern emerges in which there is still an extensive gender imbalance in archaeologists over 40. In real terms, this means that there is a lack of women in senior, managerial and professorial posts. There are a number of likely factors influencing this. There would have already been a gender imbalance in this age group due to the underrepresentation of females entering the profession in earlier years (see Moser 2007 for a discussion on the challenges of breaking into a male-dominated environment of fieldwork), in addition to the recognised challenges of the glass ceiling in archaeology resulting in further reductions in female employment as a result of career breaks which influence promotion. A further issue is that *Profiling the Profession* only assumes two gender identities, thus allowing no indication of the number of archaeologists who may conceive of their gender in ways other than the norm (as will be discussed later), and in turn, it is impossible to ascertain the correlation between this and age.

*Profiling the Profession’s* other diversity statistics demonstrate how limited our disciplinary diversity is in other areas too. 99.2 % of archaeologists are white and 98.2 % are not disabled (Aitchison and Rocks-Macqueen 2013: 99, 100). Both of these statistics are striking in their disjuncture with UK population statistics, where the 2011 census demonstrated that 86 % of the UK population are white (Office of National Statistics 2012) and 82.1 % did not have an activity-limiting health problem or disability (Office of National Statistics 2013). Indeed, where the number of white Britons is falling (Office of National Statistics 2012), the number of white archaeologists is increasing from 99 % in 2002 to 99.2 % in 2013 (Aitchison and Rocks-Macqueen 2013: 99). Whilst it is in only small numbers, it would be interesting to be able to correlate ethnicity and disability with gender in the archaeological profession to further be able to scrutinise the state of disciplinary diversity.

Elsewhere in Europe, a similar pattern is emerging with regards gender, where there are slightly more women than men in archaeology, overall, with 50.7 % being women and 49.3 % being men (Aitchison *et al.* 2014: 29). However, this masks some considerable differences in gender balance per country. In some countries such as Greece, Italy and Portugal, more than 70 % of the workforce are women, whilst in others, women make up less than 40 % of the workforce (*e.g.* Romania, Poland, Bosnia and Herzegovina, Slovakia) (Ibid.). The study did not correlate gender and age, so it is not possible to further explore the relationship between gender, age and likely seniority of posts.

Whilst the *Discovering the Archaeologists of Europe* study did not examine ethnicity, their statistics on disability demonstrate that this is as poorly represented in other European countries as it is in the UK, with only 1.1 % of those who responded about disability being disabled (Aitchison *et al.* 2014: 31). Like gender, this overall figure masks some differences amongst individual countries where, the study notes, disability may be defined differently leading to a discrepancy in how many disabled archaeologists are registered (*e.g.* the study cites that 5 % of Dutch archaeologists were disabled, whilst Denmark, Greece, Latvia and Slovakia recorded no disabled archaeologists at all) (Ibid.). This represents a fall of 0.4 % in disabled archaeologists since 2006–2008 (Ibid.: 32).

Meanwhile, Australian archaeology has also seen recent scrutiny of the professional make-up in the *Australian Archaeology in Profile* project. This comprised census-style studies undertaken in both 2005 and 2010, although these differed from those in the UK and Europe in that the survey respondents were individual archaeologists, rather than their employers (Ulm *et al.* 2013: 34). The study did not examine disability; however, gender and ethnicity were explored, and like the European and UK statistics, just over half of archaeologists (53 %) were women, but the older archaeologists, and therefore those likely to be in senior positions, were men (Ibid.: 35). In terms of ethnicity, the study shows a predominantly white population of archaeologists, with Indigenous Australians constituting only 2.3 % of respondents in 2005 and a fall in this number to only 0.8 % in 2010—in real terms, this was only three respondents (Ibid.). However, it is likely that this is a methodological issue and not the reality on the ground. As Ulm *et al.* note, ‘the recent formation of the Australian Indigenous Archaeologists’ Association, with more than 20 qualified Indigenous archaeologists …, suggests that Indigenous archaeologists are highly underrepresented in the survey’ (Ibid.: 36).

Elsewhere in the world, labour market statistics have not been as well documented as recently as in the UK, Europe and Australia. The Society for American Archaeologists led a *Discovering the Archaeologists of the Americas* pilot study in 2015 (Altschul 2014), so this picture will change for the Americas in the near future. In the meantime, however, the 1994 Society for American Archaeology census (published as Zeder’s 1997 *American Archaeologists: A Profile*) is the most comprehensive study of North America (including Canada) to date, whilst the SAA/SHA 2005 Salary Survey also provides some information about gender, age and salary (Association Research Inc. 2005). The latter demonstrates the presence of more men than women in American archaeology, with six in every ten archaeologists surveyed being male (Ibid.: c-1); however, the demographic spread matches that in Europe and Australia, where there were more women in the younger age categories (Ibid.). The study also showed explicitly that ‘male respondents were more likely to be in some types of management position—two-thirds supervised one or more people whilst only 57 % of females did so’ (Ibid.). Meanwhile, the earlier *American Archaeologists: A Profile* provides data on ethnicity, showing that 98 % of respondents were of European Heritage, 0.9 % were Hispanic, 0.6 % were Native American, 0.2 % were of Asian heritage, and 0.1 % were African American (Zeder 1997: 13).

In summary, where the statistics are recorded, the picture is bleak for ethnicity and disability, with archaeology being a majority white and non-disabled profession globally. Similarly, a global glass ceiling is evidenced, with global trends showing a gender balance until archaeologists reach their thirties or forties, and at which point the profession is dominated by male archaeologists.

These statistics show nothing new, of course. Early feminist archaeological literature (*e.g.* Conkey and Spector 1984; Gero 1985; Gero and Conkey 1991) demonstrated that archaeology is not a diverse profession. From significant female archaeologists omitted from our narratives of the discipline’s development (although cf. the recent important work by the TrowelBlazers project http://trowelblazers.com/) to the lack of gender balance in particular professional areas and the lack of diversity in terms of ethnicity and disability, we have been acutely self-conscious of these factors since at least the 1980s thanks to the post-processual critique. Indeed, a series of publications have suggested methods to address this (*e.g.* with regards to disability Phillips *et al.* 2007; Croucher and Romer 2007; Doeser *et al.* 2011 1), but as the statistics demonstrate, these have had little large scale impact in the UK and are not matched by similar projects elsewhere in Europe. So how do we address this?

We argue that, for all of their great worth, the labour market statistical studies cited above miss an important part of the archaeological demographic, students. Indeed, the studies above are not specifically designed to look at diversity in detail. Therefore, when we look to this body of archaeologists-in-training and when we question students and professionals alike about diversity, some interesting trends emerge.

In particular, we present results here from a UK wide study carried out in 2011/2012, called *Digging Diversity* (Cobb 2015). This study took the form of a short online questionnaire which was emailed to higher education institutions (HEIs) and professional archaeologists and which was posted on a number of web fora and discussion lists. There were 510 respondents, of whom 58 % were students and 42 % professionals. With an estimated 4792 people working in British archaeology in 2012 (Aitchison and Rocks-Macqueen 2013: 10), this represents about 5 % of UK professional archaeologists. This small study was very different to *Profiling the Profession* which, as noted above, is completed by employers, and thus, personal (and often undeclared) issues of diversity may be unrecorded. In contrast, the *Digging Diversity* results point to a need for a more nuanced understanding of disciplinary diversity than we can see in *Profiling the Profession* data.

For instance, participants were given the opportunity to define their own gender identity, and as a result, a small percentage (4.3 %) either chose not to define their gender at all or chose a non-normative description for their gender identity, including male transsexual, transgender, queer, gender fluid and female transgender (Cobb 2015: 237). Crucially, it was students, not professionals, who identified multiple rather than binary gender identities. This allows us to begin to unpack and challenge the binary bind that currently shapes perceptions of the makeup of our discipline. Existing labour market statistics are always given as male or female when discussing gender balance. In reality, as is well recognised in the literature, gender is culturally constructed, and in this snapshot of our archaeological culture, it is clear that gender is constructed amongst students in archaeology in multiple ways. In this respect, our accounts of the past that present crude binary divisions not only fail to reflect the realities of the past (*e.g.* see Belcher 2016 and other papers in this volume) but also fail to reflect and speak to the realities of the present. The lack of multiple gender identities in the professional respondents poses the uncomfortable question: is this lack of gender diversity real, or do we perpetuate a binary culture of practice where those who have non-normative gender identities are unable to identify themselves in this manner? That is, do we always only present our professionals with a binary check box of male and female, or worse still, do our disciplinary culture and practices restrict the performance of non-normative gender identities? We could ask the same of sexuality. *Digging Diversity* showed that just under one in five respondents (professional and student) were not heterosexual and, most worryingly, that when sexuality and professional role were correlated, those in positions of power were exclusively heterosexual.

Turning to disability, where *Digging Diversity* participants had the opportunity to state their disability, a very diverse range emerges (Table 1). As with gender, it is notable that student disabilities were much greater in range and number than professionals, and a series of issues arises from this. Many of the disabilities noted in the *Digging Diversity* study, for example, are the kind that one might not disclose to an employer who would be completing the *Profiling the Profession* or *Discovering the Archaeologists of Europe* surveys. It is likely, then, that our discipline is more diverse in terms of disability than the labour market statistics show; perhaps, there are many more archaeologists with dyslexia, dyspraxia and mental health issues, all disabilities that one can ‘get away with’ not disclosing and which carry varying degrees of social stigma, meaning that one might not want to declare these to an employer. In this manner, then, we could argue that we are complicit in perpetuating the social stigma that exists around many of these disabilities when we create and perpetuate a culture where they cannot be declared or admitted openly. The greater diversity of disabilities in students in the study likely reflects the more accepting culture in the UK higher education sector, where Disability Support Offices, or similar, at each HEI, are on hand to support students with disabilities in their studies. There must be an extent to which there are genuine elements of our profession that create practical limitations to employment in some circumstances (*e.g.* having epilepsy and not being able to drive may preclude working as a field archaeologist in a remote part of the country or being blind and thus not being able to be a field assistant on a site with steep precipices or heavy machinery), but these limitations are context-specific and do not preclude employment in other posts.

**Table 1:** The different disabilities listed by professional archaeologists and students in the 2011–2012 *Digging Diversity* study (Cobb 2015: 246)

Disability	Number of professional archaeologists	Number of students
A chronic health condition	–	1
Anxiety	–	2
Asthma	–	2
Autistic spectrum disorder (PDD)	1	–
Back injury	1	–
Chronic fatigue syndrome	1	1
Cognitive impairment	–	1
Crohn's disease	1	–
Degenerative disc disease	–	1
Depression	–	2
Diabetes	1	1
Dyscalculia	–	1
Dysgraphics	–	1
Dyslexia	6	6
Dyspraxia	3	3
Epilepsy	–	3
Golfer's elbow	–	1
Hearing impairment/loss	1	1
Hyperacusis	–	1
Ischemic heart disease	1	–
Meniere's disease	1	–
Mental illness	–	3
Missing left leg	–	1
Mobility problems	–	2
Moderate cardiac heart failure	1	–
Multiple sclerosis	–	1
Myotonic dystrophy	–	1
Myers-Irlen syndrome	–	1
Osteo-arthritis	–	1
Peripheral neuropathy	–	1
Prefer not to specify	1	–
Reduced mobility from scoliosis and injury	1	–
Sleep disorder	1	–
Stress	–	1
Tetraplegic (C6) wheelchair user	–	1
Tinnitus	–	1
Unstable angina	1	–
Vestibular disorder	–	1

Finally, when *Digging Diversity* examined ethnicity, as with gender, sexuality and disability, the picture that emerges is again more complicated than that presented in *Profiling the Profession*. A crucial difference between the two studies is that the criteria that were used for ethnicity in *Profiling the Profession* were limited to white, mixed, black or black British, Asian or Asian British, Chinese and other ethnic groups (Aitchison and Rocks-Macqueen 2013: 99). In contrast, the full list given in the 2011 census was the starting point for the *Digging Diversity* study (Table 2). Using this greater range of ethnicities enables a more nuanced analysis and crucially demonstrates that a much greater degree of ethnic diversity exists within students than professionals, with 93 % being white (Table 2). This figure is much closer to the national average (Office of National Statistics 2012). The study also demonstrates the need to move beyond simply classifying ‘white’ as one homogenous group and recognising that in this category, ethnic diversity also exists, which also has the potential to enrich the discipline.

**Table 2:** The list of ethnicities provided in the *Digging Diversity* study, after the 2011 Census categories (Cobb 2015: 242)

White: English/Welsh/Scottish/Northern Irish/British
White: Irish
White: Gypsy or Irish traveller
White: Any other White backgrounds—please specify below
Mixed/multiple ethnic groups: White and Black Caribbean
Mixed/multiple ethnic groups: White and Black African
Mixed/multiple ethnic groups: White and Asian
Mixed/multiple ethnic groups: any other mixed/multiple ethnic backgrounds—please specify
Asian/Asian British: Indian
Asian/Asian British: Pakistani
Asian/Asian British: Bangladeshi
Asian/Asian British: Chinese
Asian/Asian British: any other Asian backgrounds—please specify
Black/African/Caribbean/Black British: African
Black/African/Caribbean/Black British: Caribbean
Black/African/Caribbean/Black British: any other Black / African / Caribbean backgrounds—please specify
Other ethnic groups: Arab
Other ethnic groups: any other ethnic groups—please specify
Others, please specify

What this demonstrates is that the picture is more complicated than just those that labour market statistics allow, particularly amongst students who are not included in the labour market statistics for the UK. This is important because, as *Profiling the Profession* shows, archaeology in the UK is now a graduate profession, where 95 % of archaeologists under the age of 30 are now graduates (Aitchison and Rocks-Macqueen 2013: 12). Consequently, there is a disjuncture. British professional practice lacks disciplinary diversity, and aspects such as gender are characterised in a very normative and binary manner, but when we look to the student body, those who study archaeology are diverse in various different ways, and yet this diversity is not as clearly mirrored in the workforce. What this disparity in diversity figures between students and professionals indicates, therefore, is that there are clearly still a series of barriers that are preventing a more diverse profession. In the rest of this paper, we want to think about a way that we can move beyond these binary binds of both gender and other binaries of diversity, such as straight/gay, disabled/able bodied and white/other.

## How Do We Recentre Diversity Within Archaeology?

To address our disciplinary diversity issues, we first need to recentre diversity within archaeology. To do this, we argue, we must begin in a bottom up manner, through our pedagogy. Pedagogy (by which we mean the processes of teaching and learning) has been given some detailed consideration in recent decades, particularly in Australian and Anglo-American archaeology. Whilst a major focus of the literature has been about how we train archaeologists and the relationship between academics and professionals in this process (*e.g.* Aitchison 2004; Colley 2004; Dowson 2004; Everill 2015; Everill *et al.* 2015; Bradley *et al.* 2015; Flatman 2015), much of this literature has also provided an important source for examining diversity issues. At the heart of this literature has been a movement away from instrumentalist forms of teaching (passive student, active teacher), to approaches which recentre the student as active, and the processes of teaching and learning as politically situated and thus subjects for critical analysis in their own right (Hamilakis 2004). This movement is known as critical pedagogy and it places

an emphasis on the power of difference, on gaining new perspectives from individuals who come from a different locale in the social web of reality.... This power of difference…is key to an ever-expanding sense of criticality. This evolving criticality is dedicated to a never-ending search for new ways of seeing, for new social and cultural experiences that provide novel concepts that we can use to better understand and change the world in a progressive way*.* (Kincheloe 2008, viii)

Feminist pedagogy in archaeology has been particularly vibrant in pursuing these themes^2^, through exploring the power of different and critical approaches to teaching and learning and how they subvert gender norms, and in critically examining the production of student identities in the present as well as exploring new ways to reconsider the production of the past (Arnold 2005; Conkey and Tringham 1996; Croucher *et al.* 2014; Hendon 2006; Moser 2007; Perry 2004; Romanowicz and Wright 1996; Tomásková, 2007; Wright 1996; Wylie 2007). A smaller number of authors have considered other areas of diversity, such as ethnicity and disability, and this work has taken a different approach, focusing more on practical measures and arguing that we should change our disciplinary expectations and cultures when it comes to training students. For instance, the *Inclusive, Accessible Archaeology* project argued that we all have limitations and areas of greater ability, no matter whether we are able bodied or otherwise. Therefore, beginning by realistically assessing skills and playing to the strengths of each individual provides a productive, non-discriminatory way of practicing and of training students in the field (Phillips *et al.* 2007). Meanwhile, Croucher and Romer (2007) explored the potential to revisit some of the more exclusionary elements of excavation culture, such as the emphasis on drinking in the evenings (see also Moser 2007: 24) or the lack of segregated sleeping arrangements on training excavations, which can be exclusionary to specific religious groups and thus affect disciplinary diversity. We wholeheartedly concur with both of these approaches and with the critical pedagogy discussed above, but we argue that a more holistic approach is required if we are to truly embrace diversity in our pedagogic practices and that this should extend from the field to the classroom and to the lab and consider not simply people but also the material conditions of teaching and learning (the importance of learning spaces is already recognised in pedagogic literature, *e.g.* Oblinger 2006 (ed.)). To do this, we argue, we must turn to relational philosophical perspectives will enable us to interrogate and thus in turn to collapse the binaries of male/female, white/other and able bodied/disabled that we have demonstrated are endemic in our disciplinary culture. In particular, the use of assemblage theory can be instrumental in challenging our teaching and learning practices, whether in the lab, classroom or field.

With roots in the work of Deleuze and Guattari (1987), assemblage theory, as developed by DeLanda (2006), explores the manner in which everything is intermeshed and interconnecting along lines of flight, congealed in assemblages that are themselves more than the sum of their parts and are thus always in a state of becoming. Such an approach sees parallels with Ingold’s ideas of meshworks (2011, 2013) in considering the ontologically equal entanglements of people, things and the environment. This approach views these as interconnected and interrelated, all influencing each other as aspects of assemblages, which are themselves components of larger assemblages. The components of an assemblage may become fixed and territorialised, but equally, they may fluctuate and be de-territorialized as they influence and are influenced by other components (DeLanda 2006). Crucial to this approach is that it moves beyond an anthropocentric perspective to explore the interconnections of humans and material things as from the same ontological stance (Olsen 2010; Olsen *et al.* 2012). For instance, Bennett’s classic example of the North American blackout exemplifies this excellently. In 2003, a power cut left much of North America without electricity. In exploring who blame should be ascribed to for this event, it became clear that no one factor, human or non-human, was singularly responsible. Rather, in its failure to work, the assemblage of the power grid was highlighted as comprising, in summary (cf. Bennett 2010 for a more nuanced examination of this assemblage): people, electricity, wires, generators, trees and bush fires. No one component was responsible for the failure; it was the coming together of all of the elements, as more than the sum of their parts, which comprised the blackout. Thus, Bennett argues, the causes and consequences of the blackout are dispersed yet interconnected and inseparable as individual agents of change; ‘agency, conceived now as something distributed along a continuum, extrudes from multiple sites or many loci—from a quirky electron flow and the spontaneous fire to members of Congress who have a neoliberal faith in market self-regulation’ (Bennett 2010: 28). This example demonstrates the power of this approach to understand the manner in which humans do not exist as prior to the material world and nor are single actants, human or otherwise, responsible for single effects. Bennett’s approach highlights both the distributed and the entangled nature of affect^3^ in an assemblage.

Such a perspective is valuable as it enables us to undercut simplistic and pervasive dualities. So far, this has been applied to broader theoretical debates, such as the relationship between people and things and subject and object (*e.g.* papers in Alberti *et al.* 2013), and such approaches have already influenced archaeological interpretations of the past (*e.g.* in the edited volumes by Alberti *et al.* 2013; Watts 2013, and research by Conneller 2011; Fowler 2013; Hamilakis 2014; Harris 2014; Ingold 2013; Jones 2011; Lucas 2012; Robb and Harris 2013). However, it is less frequently used in analysing contemporary practice, with the notable exception of Lucas (2012), a work which has significantly influenced this paper.

Below, we expand on Lucas’s analysis of practice through an explicit exploration of field practice. In addition, we will explore this approach in other areas of pedagogy. Applying an assemblage theory approach to pedagogy not only incorporates current thinking on good and critical pedagogic practice (*e.g.* Hamilakis 2004; Kincheloe 2008) but goes further to consider the broader assemblages of our education, including teaching and fieldwork. And, as we will argue below, understanding the material dimensions of these assemblages is essential in two ways. Firstly, taking this approach to our pedagogy enables us to move radically away from traditional ‘banking’ models of knowledge transfer in which active teachers ‘bank’ information with passive students (Freire 1972). Instead, an assemblage approach allows us to think through the processes of teaching and learning as multiple assemblages, which are comprised of more than just an active teacher and a passive learner. In turn, we are liberated to examine how teaching and learning comprise all of the various material engagements we have as archaeologists, whether in the field or in the classroom, and how all contribute to the production of one another. Furthermore, such an approach allows us to move beyond narrow analytical frameworks that limit us to consider one area of diversity (*i.e.* gender) and one area of archaeological practice (*e.g.* just the classroom or just the profession), enabling us instead to examine how these different assemblages may be enmeshed and emerge together (Cobb and Croucher 2014), which in turn is crucial for diversifying our profession.

If we regard the multiple assemblages of teaching and learning as comprising a whole series of both material and human constituents and that all have the potential to be equally affective (as will be explored in detail in the next section), we have a useful starting point to break down the various binaries that exist surrounding both pedagogy (*i.e.* active teacher vs passive learner) and diversity. Thus, we can examine how, particularly in archaeology, the categories of archaeologists we are, be that categories of gender, sexuality, disability or categories of professional status (‘student’, ‘teacher’, ‘professional’), are not homogeneous but complicated, and emergent. Moreover, all are situated within and entwined with the materiality of doing and learning archaeology, and all emerge in various ways in relation to one another, through the practices of archaeology and the creation of new knowledge about the past. If we consider how different assemblages continually affect and constitute one another, then this transforms a view of teaching as a one way process and of students as passive repositories because it re-values learning as part of knowledge production and vice versa. In turn, this recognises students as active in the learning process and allows multiple and diverse voices to be recognised and heard, breaking the binary bind. In the next section, we explore the implications and outcomes of this through both fieldwork and the lecture theatre.

## Assembling Diversity

To illustrate the above theoretical perspectives in a tangible way, this section will discuss the assemblages of an excavation site and the university classroom. We begin with excavation and fieldwork, and in particular, we focus upon a research excavation. In most countries and continents, research excavations also train students; in part, this is for financial reasons, with research excavations frequently subsidised through either university contributions or through field schools, with varying degrees and standards in the level of training provided. The student role is usually fairly well-defined; they are trained in archaeological skills by supervisors (sometimes post-graduates, sometimes professionals) to whom they report, and they undertake a variety of tasks, rotating throughout an excavation. In terms of the general excavation outcomes, students are predominantly seen as making small contributions to the wider understanding and recording of the site, which in turn feeds into a single narrative of the project, usually written and published by the project director.

Of course, existing post-processual approaches have challenged this model, emphasising that there are multiple narratives at play on any excavation and thus highlighting the need for a democratisation of voice (*e.g.* Hodder 1997, 1999; Lucas 2001; Everill 2009). A variety of post-processual archaeologists have taken this further to work through multivocal excavation and dissemination methodologies (Chadwick 1998, 2003; Hodder 2000; Members of the Ardnamurchan Transitions Project 2012; Yamin 2012; Yamin *et al.* 2006). Some have even explored the role of materials as acting back within the archaeological process (Edgeworth 2006, 2012; Lucas 2012; Yarrow 2003, 2006, 2008), and these are approaches which speak closely to and are themselves influenced by Deleuze and Guattari and subsequent developments in the assemblage theory. However, students are often not given an explicit consideration in these works (although cf. Cobb and Croucher 2014; Members of the ATP 2012). Yet, as we have shown above, students are often central to research excavations, and it can be argued that students are often more diverse than professional archaeologists. We suggest, therefore, that examining the assemblages of teaching and learning, in the field and the classroom, provides a powerful means for deconstructing the binary bind that exists in our practices and equally provides clear insights into how we can change our practices to more fully embrace diversity.

Let us work this through an example, which is made generic here for the purposes of anonymity, but draws loosely on a series of anecdotes relayed to us by students that we teach and have encountered in our careers. Picture a research excavation, where students are being trained—this could be anywhere in the world. A trench has been opened and students are assigned to various parts of it to begin trowelling. During this process, a linear, stone, built feature emerges across the trench. After it is further cleaned and defined, the project director decides that this is the most crucial piece of archaeology on the site; this is the feature that will provide the most reliable chronological information and that may contain the most sensitive, well-preserved and diagnostic artefacts. Perhaps, there are also human remains contained within it. Immediately, students are moved off the feature. Only the director will excavate this now. Instead, the students trowel away the soil around this linear stone feature. As they trowel the soil away around it, the stone feature becomes more and more extant. The assemblage of the site, incorporating students, supervisors, the director, the trowels, the soil and the linear stone feature are all constantly affecting one another and thus constantly becoming. We can recognise this in the manner in which the trowelling by the students means that the linear stone feature emerges in relation to the contexts around it. Seeing this from an assemblage perspective, however, means that we can also recognise how the stone and the soil contexts are affective and in turn are part of the becoming of the people on the site too. Thus, as buckets, hands and trowels together remove the soil contexts, the stone feature becomes increasingly prominent on the site, directing the way people move around the trench, where buckets can sit and barrows can run. In turn, this is emphasised by the project director as they perch atop the stone feature, trowelling, brushing and photographing it, finding artefacts, noticing and recording different contexts, whilst the students move bucket after bucket of soil from around its base. In this way, the becoming of the assemblage in the present is also about the becoming of the past, and in all of this becoming, power dynamics emerge. Student identity, director identity and those of the supervisors who mediate between are all brought into being and continually territorialised in the assemblage of the trench. This is a continuous process, and so when the director decides to plan the linear stone feature and they stretch their tape across the trench, the tape bisects the contexts surrounding the feature—the tape itself divides and affects. And so does the director, as they step on recently trowelled soil, standing over the rest of students in the trench on the vantage point of the stone feature, with their planning board; the physicality of the many parts of the assemblage further brings this assemblage into being, territorialising what (parts of the archaeology) and who (which archaeologists) are important.

Some of the students (and indeed some of the staff) in this assemblage may feel that this is how it should be because they are less experienced and thus feel they have less legitimacy on the site to interpret and to act. This is reinforced by the entire assemblage of the trench: the tape that bisects the context they trowel, the emerging stone feature that increasingly stands above the soil they are removing and the shadow of the director obscuring where they are trowelling. From a pedagogic perspective, the affects of this are fundamental to teaching and learning on site because they reinforce the power dynamic of active teacher and passive student and the power dynamic which provides interpretive legitimacy to the single director whilst silencing the voices of the student body. Students are therefore rendered doubly passive, and in turn, their learning experience is negative and limited, and crucially, the diversity that, as demonstrated above, is clearly present in the student body is negated.

Indeed, in a dynamic such as that described here, the more marginalised voices in the diverse student body are further marginalised. Let us return to our hypothetical trench to explore this further. Picture, if you will, a first year undergraduate student (who, in this example, we may imagine is female, bisexual and dyslexic) trowelling on one side of the linear stone feature, who realises they have found a pit—a negative feature. Their supervisor instructs them on how to half section the pit, and as they excavate, they begin to speculate on the relationship between this pit and the upstanding feature, currently straddled by the director as they continue to plan the ‘most important’ piece of archaeology. This student brings along their own experiences to the excavation site; they are influenced by their background, their education and any prior excavation training they have received. The perspective they bring is different, unique and fresh. After a while, the student plucks up the courage to voice their interpretation to the director, the supervisors and the rest of the trench. The response is not what the student expects; the director simply laughs, and the interpretation is dismissed out of hand. The student is silenced, not just in that instance, but for the rest of the excavation, they feel their interpretations are invalidated. This has disturbing consequences. In simple terms, this limits the student learning experience; rather than thinking critically and taking the initiative, the student in question falls back into being passive, and those who witnessed the event also follow suit. On site, the message is clear: students are simply tiny cogs in a bigger machine. But this also has wider reaching affects; for the training excavation is only one part of the broader assemblage of teaching and learning in higher education (Cobb and Croucher 2014). In the classroom, the assemblage of the trench described above is in some senses replicated, with the lecturer physically removed from the cohort of students. Just as the stones, soil, tape, trowels and brushes are part of this assemblage of division and inequality on site, the benches of the lecture theatre, the PowerPoint, the handouts, the lights and more are all equally as affective—shaping and silencing the student body, positioning them to passively consume the lecture. The lesson in the field, where the student voice was silenced, is perpetuated in the lecture theatre, and the specific students in question no longer have the confidence to express their opinions or to ask questions.

Within this account, diversity is a key issue. The student in our example, being female, bisexual and dyslexic, has experienced different degrees of marginalisation throughout her lifetime for each of these different aspects of her identity, and the account above demonstrates a mechanism in which those with non-normative identities, already marginalised in wider society, may find themselves further marginalised and silenced in archaeological practice and teaching. Even though these aspects of identity may not have been an explicit factor motivating the director’s response, they nevertheless shape the experience of the student and their identity on site. We argue, then, that stifling the student voice on a training excavation, whatever the demographics or situation of the student, is to stifle diversity in a range of different ways that reach from the field to the classroom (see below), to the past, to the profession and to contemporary practice. *Digging Diversity* demonstrates that the student body is more diverse, and indeed, variations on the above fictional account have been told to us by students who are straight, gay, male, female, disabled and able bodied. The fiction we present here is more fact than many may like to believe, and consequently, the interpretations that we develop of the past are ultimately left in the hands of only a few senior archaeologists who are mostly white, have no disability, who are probably straight and who are also more likely to be male if aged over 40. Furthermore, interpretations which are unambiguous and conclusive hold more weight than what may be more accurate, ambiguous and interpretative (Gero 2007). Not only are the diversity of our interpretations stifled, therefore, but the diversity of our practices are also stymied; the message that is reinforced in the field is that the most legitimate voices in archaeology come from those white, straight, non-disabled, male senior figures at the top of the disciplinary hierarchy. In turn, this creates a climate which detracts students who do not ‘fit the mould’ from progressing in the discipline, instead perpetuating the lack of disciplinary diversity that exists in the profession.

Taking an assemblage approach then, to both our pedagogic and archaeological practice, is crucial to counter this, to break the binary bind and to diversify our profession. This is because such an approach enables us to reformulate what we do and to challenge the narratives we prioritise. On an excavation, for instance, keeping students central to the full excavation process and teaching them how to excavate the ‘most important’ areas, how to be part of the full archaeological process across the site and how to handle important features and artefacts, rather than moving them away from these, empower students and allow their diverse experiences of the world to contribute to the interpretation of the past. Equally, recognising that the material culture of excavation can be exclusionary (tape, planning frame, trench edges, the archaeology) and can itself act back to create and reinforce power dynamics allows us to re-examine how the materiality of the site can be repositioned to become inclusionary tools for the negotiation and sharing of perspectives, as sites of multivocality. In this way, the assemblage of the site emerges, not just as a multivocal interpretation of the past but as a site in which diverse people and things emerge in the present, affected positively by archaeological practice as much as by archaeological findings, by the relationship with material things and other people alike (Cobb and Croucher 2014). Further, if we accept the post-processual critique that archaeological excavations are impacted by the individuals and identities of the excavators, whether students or professionals, the diversity and demographics of the excavation will play a contributory role to the interpretations of the site. This, in turn, encourages diversity by enabling new narratives to be explored and by providing a voice to a more diverse range of participants, giving them an active place in knowledge production (Ibid.), thus enabling progression beyond existing barriers. Ultimately, because the student body is more diverse than the profession, enabling them a more prominent place and role within the excavation process will encourage a greater diversity in the discipline, allowing them access to those career paths (Cobb and Croucher 2012).

This view extends beyond the field to the various assemblages beyond the excavation site, including back in the university classroom. As teachers and students are components of the university meshwork, they act upon each other and impact each other. Rather than seeing students as passive receivers of knowledge, students are active, contributing toward the university and learning experience. For instance, within a lecture, a lecturer is influenced by the students, who act back and engage in various ways; it is not simply a case of lecturer imparting knowledge, but the questions asked by the students impact on research, through sparking new ideas and interpretations as well as influencing new ways of communicating lecturers’ research. This paper and our research into pedagogy more broadly benefit from exactly this, and in acknowledging the voices of our students, they are empowered not just in the field and the classroom or lecture theatre but beyond, in the other assemblages in which they are becoming.

This brings us to an important point for, just as acknowledging how the material elements of the trench, and of doing archaeology in the field, plays an important role in the assemblages of teaching and learning, so the materiality of the lecture theatre affects the assemblages of teaching and learning in this more traditional realm too. The negative ways in which this occurs are most obvious; the spatial configuration of projector screen, black or white board, computer terminal, microphone and the pacing lecturer at the front together create a setting for action, whilst the auditorium comprised of benches, dimmed lights and a student audience creates a setting for passivity—an assemblage of an active teacher, of a passive, homogenised student body and therefore of banking instrumentalism, which is ubiquitous in most higher education institutions. This in turn stifles diversity and perpetuates normative power relations and inequalities, as we have seen in the example above.

To challenge this, one might argue, it is either the responsibility of the lecturer to change their teaching style in a manner that asks the students to be more engaged or the responsibility of the students to engage more actively by asking questions. However, these solutions continue to reinforce a dynamic in which the student body is homogenised and beholden to the lecturer and, thus, still in many ways passive in the learning process. In contrast, through taking an assemblage approach, the power of other material components is acknowledged, along with their potential affect on academic assemblages, subverting pedagogic norms. Considering the material components of the assemblage, we can take the example of the mobile telephone in the pocket and the hand of every student or the laptop or tablet that many students will have with them. Perhaps not all students in a lecture theatre, but a likely high proportion, will be using these devices during the lecture to connect to other assemblages that extend elsewhere, outside of the lecture theatre: using Facebook, Twitter, Instagram, Snapchat and other social media, detracting from the lecture which the students are meant to be following. In many ways, the materiality of the lecture theatre is complicit in allowing students to drift from the assemblage of the lecture theatre; the wireless network to which they connect, the bench that they may ‘subtly’ hide their phone behind, the focus of all other eyes on the front to allow them to look elsewhere and the handout of lecture notes distributed by the lecturer also allow, even encourage, their attention to wander. But what if these devices become recentred as tools that render students active? An excellent example is the use of TurningPoint, a globally available piece of live polling software which allows students to use their mobile phones to vote on questions or issues posed in a PowerPoint slide and which presents the results in real time. The lecturer can pose a simple question (if x + y then what is z?), but this technology is used most effectively in what Eric Mazur calls *Peer Instruction* (Mazur 1997; Crouch and Mazur 2001). Students are given a question or scenario and a set of answers and asked to vote on what the correct answer may be. They are then given a set time to convince those around them to vote for their chosen option before the cohort is asked to vote on the same question again. This is the notion of the flipped classroom (*e.g.* Lage *et al.* 2000; Mazur 2009) but without leaving the classroom^4^. Students are engaged with the topic, passionate in arguing their perspective and enabled in all of this by their mobile device. This is just one example of the use of technology to engage. Whilst the above example involves the use of specialised software, much can be gained from simply acknowledging students that are using the internet and bringing this into the lecture. For instance, encouraging students to use live search sites on heritage databases, to explore and contribute (as a class or individually) to debates on discussion forums or social media that centre around archaeology and heritage, or to follow conference sessions being live tweeted and report back to the class, or even to encourage students to live tweet their class. In pedagogic terms, these are all interactive forms of learning that make the student active and which empower and engage them in the learning process. In philosophical terms, however, they are more than this. These are methods that ask the student to connect to other assemblages in a way that explicitly prompts them to reflect on and bring in insights from other areas of their life and the other assemblages they are entwined in. In doing so, diversity is foregrounded. By bringing encounters from external assemblages into the lecture theatre, whether archaeological (recollections of being in the field for example) or not, these will affect the student, they will affect other students and they affect the lecturer. The processes of teaching and learning, in this way, can be regarded very much as ‘in process’, in Deleuze and Guattari’s terms. Thinking through academic practice in this way can cut across binary opposites and pigeon-holed identities; breaking down the binary between students and lecturers recognises a non-linear way of learning (see Joyce and Tringham 2007), and instead, by foregrounding the assemblage(s) of teaching and learning in archaeology, ideas of binary relationships can be challenged, both in the present and in the past. This in turn allows us to contest traditional androcentric and heteronormative interpretations, providing new understandings of the past. In the present, academic practices and power structures are challenged.

At the heart of this are not just people but the assemblage that includes the physicality and materials of the lecture theatre, in the above example, the laptop, smart phone and tablet. All are active and transformative and all contribute toward shifting the loci of knowledge production from the front of the room (*i.e.* from the active teacher only) to the learner, acting back on the lecture theatre, themselves subverting traditional power dynamics and in turn further empowering students to be active. And at the heart of that empowerment, the difference and diversity of students are key to the production of new knowledge, as interpretations are inspired by different backgrounds and experiences which go against the traditional (if outdated) expected norms of the profession.

In many ways, much of what we advocate here follows the principles of critical pedagogy, as outlined earlier in the paper. Yet what these examples demonstrate is that assemblage theory liberates our pedagogic practice more than critical pedagogy alone can ever do, by encouraging us to consider the material dimensions of the processes of teaching and learning. An assemblage approach enables us to understand pedagogy as being not just about the interaction between student and teacher but about all of the various material conditions of the learning environment. The above examples have focused on technology, but we can equally consider the other material aspects of the lecture theatre and how they affect the learning experience, and how these can be used to challenge and engage, for instance, in encouraging movement within the classroom and using learning spaces differently. An assemblage approach also recognises the way in which teaching and learning is part of broader assemblages which affect the learning process. Crucially, in archaeology, this affects the way the past is understood too. In summary, an assemblage approach emphasises how the materiality of learning can also be empowering by enabling and encouraging the diverse student population to be active within the learning process, in turn challenging a homogenised and passive understanding of that process.

## Conclusion

Taking the stance toward pedagogy that we advocate here is crucial to challenge the barriers in equality and diversity that currently exist for graduates. Indeed, taking a meshwork or assemblage perspective does two crucial things. It enables us to challenge the binary categories of identity that are assigned to archaeologists, be they student or professional. It also challenges the false pigeonholing of what we actually do, demonstrating that teaching, research and fieldwork are much less separate than is traditionally presented. However, taking this approach is a brave one as it requires destabilising our current academic hierarchies which perpetuate the status quo. Those who succeed in the current parameters go on to judge others, for instance, through research councils and promotion boards. In turn, and often inadvertently, they recruit and reward in their own image, not necessarily picking individuals but types of research projects which are familiar and which conform to their experiences of high quality research. Whilst academic archaeology is affected by the wider assemblage of archaeological practice, by academic convention and by the societal norms of which it is a part, rethinking our pedagogies challenges those norms and is a starting point for change, with potentially huge, albeit long-term, impact, as our students progress through their careers to become components of the decision-making assemblages which impact on academia, archaeology and wider society. Crucially though, this is only possible by recentering the voices of the diverse student body as valuable, by valuing diversity and by empowering students who do not conform with norms to progress into the profession and academia.

What we have proposed here is a radical re-thinking of the approach we take to archaeological ontologies, based on relational philosophies. Understanding our practice as constantly emerging through assemblages where neither people nor things are ontologically prior, we can rebalance our perspectives on diversity and enable greater equity of opportunity, which challenges our binary categorisations, whether male/female, white/other, disabled/able bodied and passive learner/active teacher. Crucially, however, as well as changed attitudes, this also requires behavioural change, at a variety of levels, beginning with individuals, but also seeing institutional, national and international change in the valuation of diversity. The implications of this are wide reaching and global: such an approach plays a key role in breaking down false divisions between academia and the profession. In turn, it has the potential to break down the barriers to diversity between archaeology students and graduate archaeologists. Ultimately, if students are recognised as playing an important role in the production of an archaeological assemblage and knowledge about the past, this in turn perpetuates diversity by enabling new narratives to be told and giving a more diverse range of people a voice, thus enabling progression beyond existing barriers, and providing a bottom up framework for breaking our binary binds.

## Footnotes

1 And in the UK, an Equality and Diversity Group has just been formed within the Chartered Institute of Archaeologists to address these issues http://www.archaeologists.net/groups/equality; however, the group was only formally established in autumn 2015, so at present, there is little to report on the group’s impacts.

2 Although it should be noted that this is not explicitly under the banner of critical pedagogy, it is the authors’ assertion that such work should be considered as sharing the principles of this approach.

3 Brian Massumi, a translator of *A Thousand Plateaus*, defines affect/affection: “Neither word denotes a personal feeling (sentiment in Deleuze and Guattari). *L'affect* (Spinoza’s *affectus*) is an ability to affect and be affected. It is a prepersonal intensity corresponding to the passage from one experiential state of the body to another and implying an augmentation or diminution in that body's capacity to act. *L'affection* (Spinoza’s *affectio*) is each such state considered as an encounter between the affected body and a second, affecting, body (with body taken in its broadest possible sense to include “mental” or “ideal bodies”)” Deleuze & Guattari 1987, translated by Massumi (1987: p.xvi)).

4 The notion of the flipped classroom is a pedagogic tool in which students are tasked to research a topic outside of the classroom and come to class ready to teach their fellow cohort.

## References

[CR1] Aitchison, K. (1999). *Profiling the Profession: a survey of archaeological jobs in the UK.* York, London & Reading: Council for British Archaeology, English Heritage & Institute of Field Archaeologists. http://www.profilingtheprofession.co.uk/uploads/9/3/1/1/9311679/profiling_the_profession_1997-8.pdf. Accessed 18th May 2015.

[CR2] Aitchison K (2004). Supply, demand and a failure of understanding: addressing the culture clash between archaeologists’ expectations for training and employment in ‘academia’ versus ‘practice’. World Archaeology.

[CR3] Aitchison, K. (2009). *Discovering the archaeologists of Europe—transnational report.* http://www.discovering-archaeologists.eu/DISCO_Transnational_Report.pdf. Accessed 18 May 2015.

[CR4] Aitchison, K., & Edwards, R. (2003). *Archaeology labour market intelligence: Profiling the Profession 2002/03*. Bradford: CHNTO. http://www.profilingtheprofession.co.uk/uploads/9/3/1/1/9311679/profiling_the_profession_2002-3.pdf. Accessed 18th May 2015.

[CR5] Aitchison K, Edwards R (2008). Archaeology labour market intelligence: profiling the profession 2007-08.

[CR6] Aitchison K, Rocks-Macqueen D (2013). Archaeology labour market intelligence: profiling the profession 2011-2012.

[CR7] Aitchison, K., Alphas, R., Ameels, V., Bentz, M., Bors, C., Cella, E., Cleary, K., Costa, C., Damian, P., Diniz, M., Duarte, C., Frolík, J., Grilo, C., Initiative for Heritage Conservancy, Kangert, N., Karl, R., Kjærulf Andersen, A., Kobrusepp, V., Kompare, T., Krekovič, E., Lago da Silva, M. Lawler, A., Lazar, I., Liibert, K., Lima, A. MacGregor, G., McCullagh, N. Mácalová, M., Mäesalu, A., Malińska, M., Marciniak, A., Mintaurs, M., Möller, K., Odgaard, U., Parga-Dans, E., Pavlov, D., Pintarič Kocuvan, V., Rocks-Macqueen, D., Rostock, J., Pedro Tereso, J., Pintucci, A., Prokopiou, E. S., Raposo J., Scharringhausen, K., Schenck, T., Schlaman, M., Skaarup, J., Šnē, A., Staššíková-Štukovská, D., Ulst, I., van den Dries, M., van Londen, H., Varela-Pousa, R., Viegas, C., Vijups, A., Vossen, N., Wachter, T., & Wachowicz, L. (2014). *Discovering the archaeologists of Europe 2012–2014: transnational report*. http://www.discovering-archaeologists.eu/national_reports/2014/transnational_report.pdf. Accessed 18 May 2015.

[CR8] Alberti B, Jones A, Pollard J (2013). Archaeology after interpretation: returning materials to archaeological theory.

[CR9] Altschul, J. H. (2014). From the President. *SAA archaeological record* 14/5, p. 4–5.

[CR10] Arnold B (2005). Teaching with intent: the archaeology of gender. Archaeologies.

[CR11] Association Research Inc. (2005). 2005 salary survey conducted for the Society for American Archaeology in cooperation with Society for Historical Archaeology.

[CR12] Belcher, E. (2016). Identifying female in the Halaf: prehistoric agency and modern interpretations. *Journal of Archaeological Method and Theory, 23*(2). doi:10.1007/s10816-016-9291-1

[CR13] Bennett J (2010). Vibrant matter: a political ecology of things.

[CR14] Chadwick, A. (1998). Archaeology at the edge of chaos: further towards reflexive excavation methodologies. *Assemblage,* 3. http://www.assemblage.group.shef.ac.uk/3/3chad.htm. Accessed 18 May 2015.

[CR15] Bradley A, Geary K, Sutcliffe T (2015). Two roads: developing routes to professional archaeological practice. The Historic Environment: Policy & Practice.

[CR16] Chadwick A (2003). Post-processualism, professionalisation and archaeological methodologies. Towards reflective and radical practice. Archaeological Dialogues.

[CR17] Cobb HL, Everill P, Irving P (2015). A diverse profession? Challenging inequalities and diversifying involvement in British archaeology. Rescue Archaeology @ 40.

[CR18] Cobb H, Croucher K, Mytum H (2012). Field schools, transferable skills and enhancing employability. Global perspectives on archaeological field schools. Constructions of knowledge and experience.

[CR19] Cobb HL, Croucher K (2014). Assembling archaeological pedagogy: a theoretical framework for valuing pedagogy in archaeological interpretation and practice. Archaeological Dialogues.

[CR20] Colley S (2004). University-based archaeology teaching and learning and professionalism in Australia. World Archaeology.

[CR21] Conkey, M. W., & Spector, J. (1984). Archaeology and the study of gender. In M. Schiffer (Ed.), *Advances in archaeological method and theory*, vol. 7 (pp. 1–38).

[CR22] Conkey MW, Tringham RE, Wright RP (1996). Cultivating thinking/challenging authority: some experiments in feminist pedagogy in archaeology. Gender and Archaeology.

[CR23] Conneller C (2011). An archaeology of materials.

[CR24] Crouch C, Mazur E (2001). Peer instruction: ten years of experience and results. American Journal of Physics.

[CR25] Croucher, K., & Romer, W. (2007). Inclusivity in teaching practice and the curriculum. Guides for Teaching and Learning in Archaeology 6. Higher Education Academy. https://www.heacademy.ac.uk/sites/default/files/Number6_Teaching_and_Learning_Guide_Inclusivity.pdf. Accessed 18 May 2015.

[CR26] Croucher K, Cobb H, Brennan A (2008). Investigating the role of fieldwork in teaching and learning archaeology.

[CR27] Croucher K, Cobb H, Casella E, Rodríguez-Shadow MJ, Kellogg S (2014). Feminist pedagogy: Implications and practice. Género y Arqueología en Mesoamérica: Homenaje a Rosemary A. Joyce.

[CR28] DeLanda, M. (2006). *A new philosophy of society: Assemblage theory and social complexity*. London: Continuum Books.

[CR29] Deleuze G, Guattari F (1987). A thousand plateaus: capitalism and schizophrenia.

[CR30] Doeser, J., Dhanjal, S., Hinton, A., & Orton, C. (2011). *Diversifying participation in the historic environment workforce.* A report commissioned by the Council for British Archaeology Diversifying Participation Working Group. London: UCL Centre for Applied Archaeology.

[CR31] Dowson, T. (Ed.) (2004). Archaeological pedagogies. *World Archaeology, 36*(2), 177–309.

[CR32] Edgeworth M (2006). Ethnographies of archaeological practice: cultural encounters, material transformations.

[CR33] Edgeworth M (2012). Follow the cut, follow the rhythm, follow the material. Norwegian Archaeological Review.

[CR34] Everill P (2009). The invisible diggers: a study of British commercial archaeology.

[CR35] Everill P (2015). Pedagogy and practice: the provision and assessment of archaeological training in UK higher education. The Historic Environment: Policy & Practice.

[CR36] Everill P, Finneran N, Flatman J (2015). Training and teaching in the historic environment. The Historic Environment: Policy & Practice.

[CR37] Flatman J (2015). ‘A slight degree of tension’: training the archaeologists of the future. The Historic Environment: Policy & Practice.

[CR38] Fowler C (2013). The emergent past. A relational realist archaeology of early Bronze Age mortuary practices.

[CR39] Freire, P. (1972). *Pedagogy of the oppressed.* Harmondsworth: Penguin.

[CR40] Gero J (1985). Socio-politics and the woman-at-home ideology. American Antiquity.

[CR41] Gero JM (2007). Honoring ambiguity/problematizing certitude. Journal of Archaeological Method and Theory.

[CR42] Gero JM, Conkey MW (1991). Engendering archaeology: women and prehistory.

[CR43] Hamilakis, Y. (2004). Archaeology and the politics of pedagogy. *World Archaeology, 36*(2), 287–309.

[CR44] Hamilakis, Y. (2014). *Archaeology and the senses. Human experience, memory, and affect*. Cambridge: Cambridge University Press.

[CR45] Harris, O. J. T. (2014). (Re)assembling communities. *Journal of Archaeological Method and Theory, 21*, 76–97.

[CR46] Hendon J, Geller PL, Stockett MK (2006). Feminist perspectives and the teaching of archaeology: implications from the inadvertent ethnography of the classroom. Feminist anthropology: past, present, and future.

[CR47] Hodder I (1997). ‘Always momentary, fluid and flexible’: towards a reflexive excavation methodology. Antiquity.

[CR48] Hodder I (1999). The archaeological process.

[CR49] Hodder I (2000). Towards reflexive method in archaeology: the example at Çatalhöyük by members of the Çatalhöyük team.

[CR50] Ingold, T. (2011). *Being alive. Essays on movement, knowledge and description*. London: Routledge.

[CR51] Ingold, T. (2013). *Making. Anthropology, archaeology, art and architecture*. London: Routledge.

[CR52] Jones, A. M. (2011). *Prehistoric materialities. Becoming material in prehistoric Britain and Ireland*. Oxford: Oxford University Press.

[CR53] Joyce RA, Tringham RE (2007). Feminist adventures in hypertext. Journal of Archaeological Method and Theory.

[CR54] Kincheloe, J. L. (2008). Knowledge and critical pedagogy: an introduction. Springer Netherlands.

[CR55] Lage MJ, Platt GJ, Treglia M (2000). Inverting the classroom: a gateway to creating an inclusive learning environment. The Journal of Economic Education.

[CR56] Lucas G (2001). Critical approaches to fieldwork: contemporary and historical archaeological practice.

[CR57] Lucas G (2012). Understanding the archaeological record.

[CR58] Mazur E, Redish EF, Rigden JS (1997). Peer instruction: getting students to think in class. The changing role of physics departments in modern universities: proceedings of ICUPE.

[CR59] Mazur E (2009). Farewell, lecture?. Science.

[CR60] Cobb HL, Harris O, Jones C, Richardson P, Members of the Ardnamurchan Transitions Project (2012). The struggle within: challenging the subject/object divide on a shoestring. Reconsidering fieldwork: exploring on site relationships between theory and practice..

[CR61] Moser S (2007). On disciplinary culture: archaeology as fieldwork and its gendered associations. Journal of Archaeological Method and Theory.

[CR62] Oblinger, D. G. (ed.) (2006). Learning spaces. EDUCAUSE.

[CR63] Office for National Statistics. (2012). Ethnicity and national identity in England and Wales 2011. http://www.ons.gov.uk/ons/dcp171776_290558.pdf. Accessed 18 May 2015.

[CR64] Office for National Statistics. (2013). Disability in England and Wales, 2011 and comparison with 2001. http://www.ons.gov.uk/ons/dcp171776_296743.pdf. Accessed 18th May 2015.

[CR65] Olsen, B. (2010). *In defense of things. Archaeology and the ontology of objects*. Lanham: Altamira Press.

[CR66] Olsen, B., Shanks, M., Webmoor, C., & Whitmore, C. (2012). *Archaeology. The discipline of things*. Berkeley: University of California Press.

[CR67] Perry JE (2004). Authentic learning in field schools: preparing future members of the archaeological community. World Archaeology.

[CR68] Phillips, T., Gilchrist, R., Hewitt, I., Le Scouiller, S., Booy, D., & Cook, G. (2007). *Inclusive, accessible, archaeology: good practice guidelines for including disabled students and self-evaluation in archaeological fieldwork training.* Guides for Teaching and Learning in Archaeology 5. Higher Education Academy. https://www.heacademy.ac.uk/sites/default/files/Number5_Teaching_and_Learning_Guide_Inclusive_Accessible_Archaeology.pdf. Accessed 18 May 2015.

[CR69] Robb, J. E., & Harris, O. J. T. (2013). *The body in history: Europe from the palaeolithic to the future*. Cambridge: Cambridge University Press.

[CR70] Romanowicz JV, Wright RP, Wright RP (1996). Gendered perspectives in the classroom. Gender and Archaeology.

[CR71] Tomásková S (2007). Mapping a future: archaeology, feminism, and science practice. Journal of Archaeological Method and Theory.

[CR72] Ulm S, Mate G, Dalley C, Nichols S (2013). A working profile: the changing face of professional archaeology in Australia. Australian Archaeology.

[CR73] Wright, R. P. (Ed.) (1996). Gender and Archaeology. Pennsylvania: Penn Press.

[CR74] Watts, C. M. (Ed.). (2013). *Relational archaeologies: Humans, animals, things*. New York: Routledge.

[CR75] Wylie A (2007). Doing archaeology as a feminist: introduction. Journal of Archaeological Method and Theory.

[CR76] Yamin R, Cobb HL, Harris O, Jones C, Richardson P (2012). Through many eyes: a non-hierarchical approach to interpreting a site in New Brunswick, New Jersey. Reconsidering fieldwork: exploring on site relationships between theory and practice.

[CR77] Yamin, R., Bartlett, A. B., Benedict, T. L., Gerhardt, J., Masse, C., Milne, C. L., Raymer, L. E., & Reinhard, K. J. (2006). Once upon a time in New Brunswick, phase II/III archaeological testing and data recovery, route 18/27 (Albany Street) interchange, New Brunswick, New Jersey. Submitted to Gannett Fleming, Inc. and New Jersey Department of Transportation.

[CR78] Yarrow T (2003). Artefactual persons: the relational capacities of persons and things in the practice of excavation. Norwegian Archaeological Review.

[CR79] Yarrow T, Edgeworth M (2006). Sites of knowledge: different ways of knowing an excavation. Ethnographies of archaeological practice.

[CR80] Yarrow T, Kanppett C, Malafouris L (2008). In context: meaning, materiality and agency in the process of archaeological recording. Material agency.

[CR81] Zeder MA (1997). The american archaeologist: a profile.

